# Organic/Inorganic Nanocomposites Based on ‘Three Pillars’ (Organic Compounds, Metal Nanoparticles, and Carbon Nanomaterials)

**DOI:** 10.3390/ijms26146578

**Published:** 2025-07-09

**Authors:** Tamara Basova

**Affiliations:** Nikolaev Institute of Inorganic Chemistry SB RAS, 3 Lavrentiev Pr., 630090 Novosibirsk, Russia; basova@niic.nsc.ru

A wide variety of organic molecules, ranging from simple aromatic molecules to complexes with organic ligands and polymers, offer the possibility of creating both simple and complex structures with diverse physicochemical properties. The study of organic/inorganic composite materials is an increasingly popular and interdisciplinary field within materials science and engineering [1,2]. Materials based on “three pillars”—organic compounds, metal nanoparticles, and carbon nanomaterials—are of special interest due to their wide range of practical applications in organic electronics and medicine. Metal nanoparticles have a high surface-to-volume ratio and exhibit catalytic activity and antibacterial properties. Carbon nanostructures include a diverse range of materials such as carbon quantum dots, nanotubes, nanofibers, and graphene. These three components serve as the foundation for creating hybrid and composite materials that combine synergistic physical and chemical properties. Organic/inorganic nanocomposite materials are attractive for researchers from both the perspective their structure and properties and from the perspective of their potential applications in various fields, e.g., in sensors for monitoring pollutants in water, food, and biological media, medical diagnostics and therapy, nanotechnology, and energy production.

Articles published in this Special Issue include cutting-edge research on new synthetic procedures for creating functionalized organic compounds and their composites, as well as on their diverse properties and applications in important areas such as chemical sensing, cosmetic production, and medicine.

Two research articles in this Special Issue focus on the synthesis and properties of two important classes of organic compounds: naphthyridine derivatives and borosilicates. Naphthyridines, particularly the 1-oxo-2,7-naphthyridine derivative, have shown great potential in medicine due to their biological activity, and they may be used to treat inflammatory immune disorders and chronic inflammation [3]. Borosilicates are important hybrid organosilicon compounds that are widely used as building blocks and starting materials for further organometallic synthesis.

The study by Sirakanyan et al. [4] focuses on the synthesis and rearrangement of new 1,3-diamino-2,7-naphthyridines and 1-amino-3-oxo-2,7-naphthyridines. They discovered that the rearrangement reaction was influenced by both the substituent at the seventh position of the 2,7-naphthyridine ring and the nature of the cyclic amine at the first position. This influence was primarily due to steric effects. Expanding the scope of the rearrangement allowed for a deeper understanding of its reaction mechanism and provided an opportunity to develop a novel, selective method for the synthesis of 2,7-naphthyridine derivatives.

Frydrych et al. [5] conducted research on a novel type of borasilsesquioxanes synthesized through a condensation process. They explored their behavior in catalytic hydrosilylation reactions with silanes, siloxanes, and silsesquioxanes. They reveal that the mono- and diethynylphenylborasilsesquioxane derivatives exhibit greater reactivity compared to their vinyl counterparts. Through the analysis of the various stages of borasilsesquioxane decomposition, they also discover that the structure of borasilsesquioxanes underwent transformation during the pyrolysis process, resulting in the formation of polymeric compounds.

Wiśniewska et al. [6] conducted a study on the use of an innovative amber-based composite in the field of cosmetics. To achieve this, they developed a three-component natural composite consisting of amber, diatomite, and Phytokeratin^TM^ (a hydrolyzed plant protein) using a mechanochemical synthesis process. Their objective was to enhance the release of biologically active substances (succinic acid and Phytokeratin^TM^) in an aqueous solution. The resulting composite was successfully integrated into environmentally friendly cosmetic formulations, including a solid shampoo bar and a nail conditioner.

The other two articles in this Special Issue focus on the investigation of MXene derivatives and their composite materials. MXenes are a class of metallic carbon/nitride materials with 2D nanostructures that have found wide applications in various important areas, such as energy storage, chemical sensing, catalysis, and biomedicine [7].

Li et al. [8] explore the potential of using photoactive AuNP/MXene–BiOCl Moiré Superlattice Nanosheets in a photoelectrochemical (PEC) biosensor for detecting Protein Kinase Activity (PKA). AuNP/MXene–BiOCl offered a high photo-to-current conversion efficiency, excellent electrical conductivity, and a large surface area for immobilization, which increased the number of kemptide molecules that could be loaded and also facilitated faster spatial charge separation. The newly developed PEC sensor demonstrated exceptional performance, with a remarkable sensitivity of 0.0029 U mL^−1^ to PKA. This achievement opens up new possibilities for drug discovery and the diagnosis of diseases associated with kinases.

Wang et al. [9] developed a composite membrane made from porous MXene nanosheets with etched nanopores combined with cellulose nanofibers (CNF), and they tested its efficacy in achieving high-performance osmotic power generation. The etched nanopores on MXene sheets act as interconnected cationic nanochannels, significantly increasing the ion flux and selectivity to cations in the composite system. At neutral pH and room temperature, the MXene/CNF composite membrane demonstrated a maximum output power density of 0.95 W·m^−2^ with a 50-fold KCl concentration gradient.

Gold nanoparticles (AuNPs) have garnered significant attention in the medical field due to their exceptional physicochemical properties and remarkable biocompatibility. They also exhibit customizable optical properties, making them an ideal candidate for targeted drug delivery and imaging applications. Additionally, GNPs can be readily functionalized with a variety of molecules, providing a versatile platform for a wide range of therapeutic applications [10].

Farhana et al. [11] conducted a groundbreaking study that identified novel therapeutic targets for AuNPs in the field of cancer biology. Synthesized spherical AuNPs with an average size of 28.3 nanometers were found to be highly effective in treating breast cancer cells. The study reveals that AuNPs can inhibit the production of interleukin-6 mRNA/protein by upregulating miR-26a-5p and deactivating the RelA and NF-κBp50 transcription pathways in cancer cells. These findings provide valuable insights into the molecular mechanisms through which gold nanoparticles protect against breast cancer.

Sergeevichev et al. [12] conducted a study to assess the biocidal effect and cytotoxicity of the Ag/Ir and Ag/Au film heterostructures deposited onto implant biomaterials. The researchers used state-of-the-art implant materials (a Ti alloy and CFR-PEEK polymer, 30% carbon fiber), which were functionalized with film heterostructures consisting of Ir or Au sublayers with a Ag-based antibacterial component deposited on top. They demonstrated that the activity of Ag/Ir heterostructures can be attributed to the high release rates of Ag^+^, which result in the rapid inhibition of *P. aeruginosa* growth within 2–4 h. In contrast, the inhibition of *P. aeruginosa* and *S. aureus* growth by Ag/Au heterostructures occurs more slowly, taking 6 h or more. The antibacterial activity appeared to be due to the combined action of Ag^+^ and Au^+^ ions.

We would like to extend our appreciation for the outstanding quality of work all the authors who submitted their manuscripts to our Special Issue provided. We hope that this Special Issue will provide a forum for discussion between researchers in the field of organic/inorganic nanocomposite materials.
